# Nutritional Status and Dietary Intakes of a Community of Rural Women in Bárcena Villa Nueva, Guatemala: A Small-Scale Observational Study

**DOI:** 10.3390/nu18030512

**Published:** 2026-02-02

**Authors:** Sara Basilico, Angeliki Sofroniou, Maria Vittoria Conti, Paola Dieguez, Hellas Cena

**Affiliations:** 1 Laboratory of Dietetics and Clinical Nutrition, Department of Public Health, Experimental and Forensic Medicine, University of Pavia, 27100 Pavia, Italy; mariavittoria.conti@unipv.it (M.V.C.); hellas.cena@unipv.it (H.C.); 2 Department of Food and Drug, University of Parma, 43121 Parma, Italy; angeliki.sofroniou@unipr.it; 3 The Missionary Sisters of the Sacred Heart of Jesus, Cabrini Foundation, Zone 6, Guatemala City 01006, Guatemala; nutricion.cabrini@gmail.com; 4 Clinical Nutrition Unit, Istituti Clinici Scientifici Maugeri, Istituto di Ricovero e Cura a Carattere Scientifico, 27100 Pavia, Italy; 5 Italian Institute for Planetary Health, 00168 Rome, Italy

**Keywords:** developing countries, obesity, nutrition, women, dietary diversity, malnutrition double-burden

## Abstract

**Background/Objectives**: Rural communities in Guatemala face a growing double-burden of malnutrition. Women of reproductive age are a key population to address, as their health and nutritional status influence not only their own well-being but also that of their children and families. However, they often experience greater exposure to nutritional risks due to gendered inequalities in access to resources, education, and health care. This small-scale observational study aimed to describe the dietary habits and nutritional status of a sub-group of women living in a rural area of Bárcena Villa Nueva, Guatemala. **Methods**: An observational study was conducted between March and April 2025 among women aged ≥18 years from two rural communities. Dietary data were collected through structured interviews, 24 h dietary recall (24-hR), and a validated food frequency questionnaire (FFQ). Anthropometric and biochemical measurements were also collected. **Results**: A total of 22 women were included (mean age: 41.3 ± 16.3 years). The prevalence of obesity and central obesity was 45.5% and 86.0%, respectively. Quantitative dietary assessment based on the 24 h recall showed a mean energy intake approximately 35% higher than the recommended values, with a high contribution from fats and carbohydrates and an excessive sodium intake. In contrast, intakes of potassium, zinc, and folic acid were below the recommended levels. The qualitative analysis of the food frequency questionnaire indicated a dietary pattern characterized by high consumption of carbohydrates, animal-based protein sources, traditional energy-dense foods, and ultra-processed products, alongside a limited intake of vegetables and fruits. Datary diversity was low (4.9, SD: 1.1). **Conclusions**: This small-scale observational scale study provides a preliminary overview of dietary patterns and nutritional status among women living in a rural community in Guatemala. Although the findings are not generalizable, they suggest the coexistence of excessive energy intake, suboptimal micronutrient intake, and low dietary diversity. These results underscore the need for further research using larger and more representative samples and may help inform the development of context-specific nutrition education initiatives in similar underserved settings.

## 1. Introduction

Guatemala faces a complex nutritional landscape characterized by the coexistence of undernutrition and overnutrition, a phenomenon known as the double burden of malnutrition [1,2]. Despite being a country rich in biodiversity with significant agricultural resources [3], chronic undernutrition remains widespread, especially among Indigenous and rural populations, where childhood stunting reaches rates as high as 70% [4]. At the same time, a marked rise in overweight, obesity, and non-communicable diseases has been observed among adults, particularly women, whose obesity prevalence increased by over 22% since the 1990s, reaching 32% nationally [5,6]. These trends reflect ongoing socioeconomic changes and shifts toward Westernized dietary patterns driven by urbanization, increased access to ultra-processed foods, and limited availability of nutrient-dense options [7,8].

Women of reproductive age represent a critical group in this context [9,10]. They play key roles as caregivers and food preparers within households, influencing family dietary patterns and intergenerational health outcomes [11,12]. Yet, they are disproportionately exposed to nutritional risks due to gender-based disparities in access to education, health services, and economic resources [9,10]. Although several studies have documented these trends at the national level, limited research exists on rural women living in peri-urban or transitioning areas, where traditional food environments coexist with industrialized food supply chains.

Bárcena, located in the municipality of Villa Nueva, is one such setting. It combines rural characteristics with rapid industrial development and urban influence, yielding a unique environment for potential dietary and nutritional transitions. Preliminary field observations conducted by the Cabrini Foundation have suggested heightened risk factors among women in this area, including early onset of overweight, low dietary diversity, and dependence on inexpensive energy-dense foods. However, to date, there is no published data characterizing the nutritional status or dietary habits of women in this community.

This small-scale observational study aims to fill this gap by describing the anthropometric measurements, biochemical parameters, and dietary composition of group of adult women from two rural communities in Bárcena Villa Nueva. The findings are intended to inform the design of tailored nutrition interventions and to contribute to the broader understanding of the double burden of malnutrition in rural Guatemala.

## 2. Materials and Methods

### 2.1. Study Overview and Partnership

This small-scale observational study was conducted within the framework of a formal Research Collaboration Agreement signed in February 2025 between the University of Pavia (Italy) and the Cabrini Foundation (Guatemala). The agreement regulated scientific collaboration activities related to study design, data analysis, and dissemination, without establishing shared governance, joint appointments, or financial dependency between the two institutions. Researchers from the Laboratory of Dietetics and Clinical Nutrition (LDNC) of the University of Pavia were responsible for study conceptualization, protocol development, data analysis, interpretation of results, and manuscript preparation. No University of Pavia personnel were involved in participant recruitment or data collection activities, nor were they physically present in the study setting. Participant recruitment and data collection were conducted exclusively by trained dietitians affiliated with the Cabrini Foundation in Guatemala, who operated independently from the academic research team.

#### 2.1.1. Laboratory of Dietetic and Clinical Nutrition

The LDNC is a multidisciplinary research group with national and international expertise in the fields of dietetics and clinical nutrition, lifestyle medicine, and personalized nutrition. The group’s research has focused on malnutrition both undernutrition and overnutrition related to non-communicable diseases, with particular attention to vulnerable populations (children and adolescents, women of childbearing age, and the elderly). Within this study, LDNC researchers acted as scientific leads and were responsible for protocol development, data analysis, interpretation, and scientific reporting.

#### 2.1.2. Cabrini Foundations

Cabrini Foundations is a non-governmental organization led by the Missionary Sisters of the Sacred Heart of Jesus, a religious institute founded by Saint Frances Cabrini. The Foundation provides a wide range of healthcare and community services and maintains affiliations with Italian universities, including the University of Pavia, while operating as an independent entity. Within this study, dietitians affiliated with the Cabrini Foundation were responsible for participant recruitment and on-site data collection.

### 2.2. Study Setting

#### 2.2.1. Communities’ Description

The study was conducted in two neighboring communities, Bárcena and Colonia 20 de Octubre, located in the municipality of Villa Nueva, Department of Guatemala. Villa Nueva is a densely populated and economically dynamic municipality within the metropolitan area of Guatemala City, characterized by a mixed economy dominated by industry, commerce, community services, and construction. While employment is largely linked to industrial and commercial activities, small-scale agriculture and livestock production persist in peripheral communities such as Bárcena and Colonia 20 de Octubre. According to the 2018 National Census, Villa Nueva has 433,734 inhabitants, with women representing 51.8% of the population.

Bárcena, the primary study area, is one of the most populated localities within the municipality, with an estimated 16,211 inhabitants. It is a semi-urban community located in Zone 3 of Villa Nueva, approximately 4 km from the municipal center, and has undergone progressive urbanization from a historically agricultural setting to a mixed rural–urban landscape. The area includes residential zones, small markets, schools, local health posts, and community facilities, and benefits from its proximity to the Ruta al Pacífico, which facilitates daily commuting to Guatemala City.

Colonia 20 de Octubre is a smaller residential settlement within the broader Bárcena area (Zone 3), composed mainly of family households clustered around local infrastructures such as a primary school, community gathering spaces, and small shops. Like many peri-urban colonias in Villa Nueva, it is characterized by dense settlement patterns, strong neighborhood networks, limited formal services, and reliance on informal labor and daily commuting for employment.

Together, these communities represent typical rural–urban transitional settings in Guatemala, where traditional dietary patterns coexist with increasing exposure to urban food environments and ultra-processed foods. Their socioeconomic profile and limited access to health and nutrition services make them particularly relevant for investigating the double burden of malnutrition among women.

#### 2.2.2. Food Environment Context

The food environment in Bárcena and Colonia 20 de Octubre reflects the broader urban–peri-urban food system of Villa Nueva and the Guatemala City metropolitan area. Rapid urban expansion and the progressive loss of agricultural land have shaped a food environment dominated by small local stores (“tiendas”), informal markets, and periodic vendors, with limited presence of large supermarkets. Most households rely on nearby small retailers or central markets in Villa Nueva and adjacent urban areas, where fresh produce, staple foods (e.g., maize and beans), and processed products coexist.

Although traditional staples such as maize tortillas and beans remain culturally central and widely available, nutritionally diverse and healthy food baskets are often less affordable and inconsistently accessible for low-income households. Economic constraints related to informal and unstable employment limit purchasing power and favor the consumption of low-cost, energy-dense, and nutrient-poor foods, including ultra-processed products, which are widely available and relatively inexpensive compared with fresh fruits and vegetables.

Dietary behaviors are further influenced by living conditions, education, and cultural factors. Traditional dietary patterns coexist with a growing reliance on convenience foods and sugar-sweetened beverages, a trend commonly observed in rapidly urbanizing settings. In this context, limited access to healthy foods and nutrition information contributes to the coexistence of undernutrition and increasing overweight and obesity among women, framing the food environment in which this study was conducted.

### 2.3. Study Population

The target population included women aged over 18 years, with no medical conditions that could interfere with regular eating habits, who were affiliated with two local communities (20 Octubre and Bárcena). All participants were members of the Cabrini Foundation community network. Only women who provided written informed consent were included in the study.

Participants were recruited through the Cabrini Foundation community network, one of the few stable and accessible health and social outreach structures in the area. This non-probabilistic, convenience sampling approach reflects the exploratory nature of this small-scale observational study, which was not intended to generate a population-representative sample of Bárcena but rather to provide preliminary insights to guide future, larger-scale investigations. Because women engaged with the Cabrini Foundation are often more involved in community health and support programs, the sample may over-represent individuals with greater health awareness or more regular participation in such services, potentially influencing their dietary behaviors and anthropometric characteristics compared with women less connected to these community networks.

### 2.4. Ethical Considerations

In the region where the study was conducted, formally constituted ethics committees were not available to evaluate research protocols at the time of data collection. In accordance with the local regulatory framework, ethical approval for the study was therefore granted by the Cabrini Foundation, based in Villa Nueva, Guatemala. Prior to implementation, the full study protocol also underwent internal review by the research team at the University of Pavia to ensure compliance with internationally recognized ethical standards, including participant protection, risk minimization, voluntary participation, and appropriate informed consent procedures. We explicitly acknowledge that this internal review does not replace independent ethics committee review. The absence of a formally constituted independent ethics committee/IRB in the study area at the time of data collection is therefore a procedural limitation of this study. All participants provided informed consent prior to enrolment. The consent form clearly described the study objectives, procedures, and potential implications. For participants who were unable to read or write, a trained practitioner provided a complete verbal explanation of the consent document to ensure full comprehension. In these cases, informed consent was documented through the practitioner’s signature together with the participant’s fingerprint, in accordance with local ethical and legal practices. The study was conducted in accordance with the core principles of the Declaration of Helsinki (risk minimization, voluntary participation, and confidentiality) and adhered to all relevant regulations concerning the privacy, confidentiality, and protection of participants’ data. Although the Cabrini Foundation is a religiously affiliated organization, participant recruitment and data collection were carried out exclusively by trained dietitians and community health workers who did not hold religious roles or positions of authority over participants. Participation in the study was entirely voluntary and was not linked to access to healthcare or social services provided by the Foundation. All participants were explicitly informed that refusal to participate or withdrawal from the study would have no consequences for their access to any services, thereby minimizing any risk of coercion or undue influence.

### 2.5. Data Collection

The data collection process was carried out throughout April 2025 among women of Cabrini Foundation community network. A case report form (CRF), adapted from the validated “Manual de instrumentos de evaluación dietética” developed by the Instituto de Nutrición de Centro América y Panamá (INCAP) in Guatemala [13], was used to collect information on the women and their households, to assess their dietary habits, and to record biochemical data. Data were collected by two qualified dietitians volunteering at the Cabrini Foundation community center. Prior to study initiation, the field staff received protocol-specific training on all study procedures and on the administration of the CRF and dietary tools. Standardized written instructions were used for interviews and measurements. Before analysis, datasets were checked for completeness and plausibility; when inconsistencies or missing values were identified, clarifications were requested from the field team.

#### 2.5.1. Sociodemographic Information

A structured interview was used to collect socio demographic information (ethnicity, marital status, education level, occupation, household composition).

#### 2.5.2. Anthropometric Measurements

A trained local dietitian conducted the anthropometric assessments of the enrolled women. Body weight was measured using a calibrated digital scale, with participants asked to remove their shoes and wear light clothing. Measurements were recorded to the nearest 0.1 kg. The scale also reported the percentage of fat mass and lean mass. Height was measured using a portable stadiometer and recorded to the nearest centimeter. Waist circumference (WC) was measured at the midpoint between the lower rib and the iliac crest to the nearest 0.5 cm, while hip circumference (HC) was measured at the widest part of the buttocks to the nearest 1.0 cm. Body Mass Index (BMI) was calculated as weight expressed in kilograms divided by height in meters squared, and participants were categorized based on standard BMI cutoffs: underweight (BMI < 18.5 kg/m^2^), normal weight (BMI 18.5–24.9 kg/m^2^), overweight (BMI 25–29.9 kg/m^2^), and obesity (BMI ≥ 30 kg/m^2^). Waist-to-height ratio (WHtR) was computed as WC divided by height, and waist-to-hip ratio (WHR) as WC divided by HC, with all measurements expressed in centimeters. A WHtR value of 0.59 or greater was used as a marker of central adiposity [14], while a WHR value of 0.85 or higher was considered indicative of increased cardiometabolic risk [15].

#### 2.5.3. Dietary Composition Assessment

A semi-structured 24 h dietary recall (24-hR) was administered to women participating in the study to estimate energy and nutrient intake. The tool was developed by the Institute of Nutrition of Central America and Panama (INCAP) for both household food consumption and individual dietary intake assessments was employed in this study [13]. As participants typically prepare meals for the entire household rather than for themselves individually, dietary intake was assessed at the household level. Individual energy and micronutrient intakes were then estimated from household data by proportionally allocating foods according to age- and sex-specific consumption patterns within the household. This approach reflects culturally appropriate practices and has been used in similar community-based nutritional studies. To enhance the accuracy of fluid intake estimation, specific follow-up questions regarding all types of beverages (including water) were included, alongside the use of measuring cups. Additional questions addressed the consumption of packaged foods and the cooking methods used (e.g., frying, boiling, steaming, sautéing).

In addition, a validated food frequency questionnaire (FFQ) was used to qualitatively describe habitual food consumption patterns and food group frequencies of each participant [16]. Individual surveys were administered separately to avoid biasing the respondents. Personal data was collected, and then the frequency of each food item, including the portion size, was recorded. Standard measurements of certain foods were used and measured portions.

Dietary intake data from the 24-hR was analyzed for energy, macro- and micronutrient. The “Central American Food Composition Table” by INCAP [17] was used to estimate food composition, providing data on energy (kcal), total proteins (g), total fats (g), saturated fats (g), polyunsaturated fats (g), cholesterol (mg), carbohydrates (g), fiber (g), calcium (mg), sodium (mg), potassium (mg), magnesium (mg), phosphorus (mg), zinc (mg), iron (mg), vitamin A (IU), thiamin (mg), riboflavin (mg), niacin (mg), vitamin B6 (mg), vitamin B12 (µg), folic acid (µg DFE), and vitamin C (mg).

#### 2.5.4. Dietary Quality Assessment

Diet quality was evaluated with the Minimum Dietary Diversity Index for Women (MDD-W), a food group diversity indicator that has been shown to reflect one key dimension of diet quality: micronutrient adequacy, summarized across 11 micronutrients [18]. This is a dietary diversity indicator developed exclusively for women, because of nutritional vulnerability affecting them during reproductive age [19]. For all these reasons, the MDD-W [18] was evaluated on women, based on 24-hR. According to the MDD-W protocol [18], foods were classified into ten groups: (1) grains, white roots and tubers, and plantains; (2) pulses; (3) nuts and seeds; (4) dairy; (5) meat, poultry and fish; (6) eggs; (7) dark green leafy vegetables; (8) other vitamin A-rich fruits and vegetables; (9) other vegetables; (10) and other fruits. Food groups included in the MDD-W index mostly suggested diet quality with adequate micronutrient intake considering the most important micro- nutrients [18]. For each food group, a dichotomous variable was used: “1” for women who consumed any food item in the group at least one time per day and “0” for those who did not consume any food within that food group. The MDD-W index was calculated by summing up the number of food groups consumed. The adequacy in micronutrient intake was reached when the index was equal to or higher than five.

#### 2.5.5. Biochemical Data

A subgroup of enrolled women underwent a fasting blood draw in the morning to assess the following biomarkers: blood glucose, total cholesterol, triglycerides, seric creatinine, seric uric acid and Blood Urea Nitrogen (BUN). Biochemical analyses were performed and processed by a private, local, independent clinical laboratory (SALUD DIGNA), which is not affiliated with the Cabrini Foundation. Analyses were conducted according to the laboratory’s routine internal quality-control procedures. Reference ranges and abnormal-value flags were those reported by the laboratory in the official test reports and were used for descriptive interpretation. Women participating in the project received a voucher covering 100% of the costs.

### 2.6. Statistical Analysis

Descriptive and exploratory analyses were performed to summarize the baseline characteristics of the enrolled women. Categorical variables were reported as counts and percentages. Continuous variables were summarized as means ± standard deviation (SD) or as medians and interquartile ranges (IQR), as appropriate, based on data distribution. All analyses were conducted at the individual level.

Dietary intake in terms of energy, macro- and micronutrients was calculated from the 24 h dietary recalls collected during the nutritional assessment.

An optimal dietary intake was estimated for each participant. This estimation was based on the following parameters, derived from the INCAP daily nutritional recommendations [20] to ensure maximal relevance to the study population. Ideal Body Weight (IBW) Calculation: The IBW used to estimate the Basal Metabolic Rate (BMR) was determined using a reference Body Mass Index (BMI) of 22 kg/m^2^ consistent with the WHO/FAO/UNU recommendations for calculating energy needs in adults [21], as adopted by INCAP [20]. A Physical Activity Level (PAL) of 1.55 (representing ‘light activity’) was uniformly applied. This value was selected because all study participants reported a predominantly sedentary lifestyle, aligning with the reference level for adult women in the INCAP guidelines for non-strenuous activity [20]. The final optimal energy intake was calculated to be approximately 1900 kcal/day on average. The subsequent dietary reference values for macro- and micronutrients were strictly derived from the official INCAP daily nutritional recommendations for adult women [20].

Observed intakes obtained from the 24 h recalls were descriptively compared with the corresponding reference values to evaluate deviations from the estimated optimal dietary composition.

Given the small-scale observational design and the limited sample size, no inferential statistical tests or group comparisons were performed. The statistical analysis was therefore intentionally limited to a descriptive and exploratory framework. Statistical computations were performed using R (version 2025.05.0 + 496) statistical software. Missing data were handled using a complete-case approach, and no imputation was applied.

## 3. Results

Recruitment lasted throughout the month of March 2025, and the data collection was completed in April 2025. Twenty-two women who approved and signed the informed consent were included in the study.

### 3.1. Overall Sample Description

The mean age of participants was 41.3 (16.3) years. Overall, the educational level was moderate, with 41% of women having completed middle school. Most participants were housewives (95%) and were married or living with a partner. The most common household size included 4–5 members, and 77% of women lived with one or two children (Table 1).

### 3.2. Anthropometrics Measurements

Anthropometric characteristics of the study population are reported in Table 2. Among the overall sample, 22.7% [95% CI: 4.7; 40.7] presented overweight, while 45.5% [95% CI: 24.7; 66.3] presented obesity. A WHtR ≥ 0.59 was found in 86% (*n* = 19) of the sample indicating presence of central obesity, while almost all the sample (*n* = 21; 95%) showed a WHR ≥ 0.85, highlighting a high cardiometabolic risk.

### 3.3. Eating Habits and Dietary Composition

#### 3.3.1. 24-hR

Based on the 24-hR, the most frequently reported cooking method was boiling, with a median frequency of 3.25 times per week [IQR 2.5–3.25], followed by frying, reported 2 times per week [IQR 1.5–2], with a maximum of 4.5 times per week. Other cooking methods reported included roasting (59% of the sample), steaming (41%), and sautéing (14%). Table 3 presents the comparison between the mean intakes obtained from the 24-hR (*n* = 22) and the INCAP daily nutritional recommendations [20]. Overall, energy intakes appeared to be above the recommended values for adult women [20]. Although the relative contribution of macronutrients to total energy intake was within or slightly below the recommended proportions for a 1900 kcal reference diet, the absolute intakes (in grams and corresponding kilocalories) were considerably higher. An excessive consumption of carbohydrates, proteins, and total fats, emerged, while the consumption of fiber intake was below the optimum (30 g). Regarding minerals, calcium, potassium and zinc intakes were below recommendations. Between the assessed vitamins, vitamin A, vitamin C and folic acid intakes were below recommendations.

#### 3.3.2. FFQ

The FFQ analysis revealed a mixed dietary pattern, reflecting a traditional base typical of rural or semi-urban Guatemalan populations, characterized by a high intake of complex carbohydrates, primarily rice, maize, and pasta, combined with low vegetable variety and a high reliance on animal-source proteins, mainly chicken and eggs. At the same time, the consumption of packaged ultra-processed foods indicates an ongoing nutrition transition toward more Westernized dietary patterns. Regarding carbohydrate sources, the entire sample (100%) reported regular consumption of rice (boiled or fried), corn tortillas, and *fideos*, a locally consumed pasta similar to spaghetti. Other starchy foods such as *plátano* and potatoes were reported by 89% of participants, while baked products such as bread were consumed by 53%. Among legumes, beans were consumed almost daily in various traditional preparations, particularly *frijoles parados* (68%), whole boiled beans typically served with broth, and *frijoles volteados* (53%), a refried bean preparation mashed with added fat, commonly consumed as a spread or side dish.

Animal protein sources were dominated by eggs and chicken (84%), with chicken frequently consumed fried (74%), followed by fresh fish (74%) and beef (68%). A high intake of processed meats was also observed, including sausages (74%) and fried pork rind (*chicharrones*, 63%). Dairy consumption was limited, with cheese consumed by 16% of the sample, while cow’s milk was reported by 68%, often in traditional preparations such as atol, a traditional beverage made from fresh maize, milk, sugar, and cinnamon.

Vegetable intake was largely centered on a limited number of items, mainly carrots (84%) and local vegetables such as *guisquil* (chayote-like squash; 74%) and *guicoy* (round zucchini, often stuffed with meat or cheese; 58%). Green leafy vegetables were less frequently consumed (16%), primarily chard and spinach, usually prepared cooked in soups or stews. Fruit intake mainly included tropical fruits, with papaya (63%), watermelon (53%), bananas (53%), and mango (42%) being the most commonly reported. Traditional energy-dense foods such as tamales and pupusas were consumed by all participants (100%). Tamales are steamed maize-based dough preparations, usually filled with meat, sauces, or vegetables and wrapped in banana or maize leaves, while pupusas are thick maize tortillas stuffed with cheese, beans, or meat and cooked on a griddle. Additionally, atol was reported by 80% of the sample. Concurrently, the FFQ captured the consumption of Western-type foods, including frozen pizzas (68%), instant soups (53%), packaged ice creams (37%), and ready-made sauces such as mayonnaise (53%). Among fats, vegetable margarines were the most commonly used (70%). The intake of sugar-sweetened beverages was also high, particularly cola-type soft drinks, flavored sodas, and sugar-added fruit juices (79%).

### 3.4. Dietary Diversity

Total mean MDD-W index was 4.9 (SD: 1.1) and micronutrient adequacy (MDD-W index ≥ 5) [18,19] was achieved by 9 women constituting 40.9% of the whole sample. Table 4 shows the reported consumption of different food groups that were used for the MDD-W index calculation.

**Figure 1:**
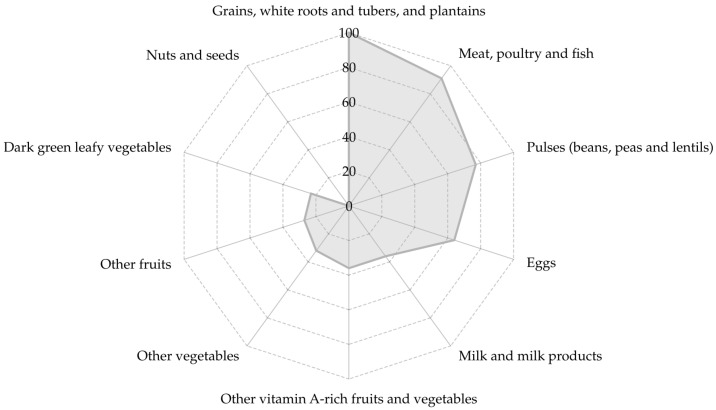
The most frequently consumed food groups of the MDD-W [18]. The figure shows the most frequently consumed food groups considered to evaluate the minimum dietary diversity index for women (MDDI-W) reported as percentage.

The most frequently consumed groups were “grains, white roots and tubers, and plantains” (100.0%), followed by “meat, poultry and fish” (90.9%), “pulses” (77.3%), “eggs” (63.6%). While “nuts and seeds” (0.0%), “dark green leafy vegetables” (22.7%) and “other vegetables” (36.4%) were least frequently consumed, Figure 1.

### 3.5. Biochemical Data of a Subgroup of the Sample

The biochemical data were collected among a subgroup of 11 participants (50% of the sample) and are shown in Table 5.

By correlating the biochemical data with the value of abdominal circumference, it was possible to identify the presence of metabolic syndrome in 5 out of 11 women [22].

## 4. Discussion

This small-scale observational study aimed to provide baseline data to guide the development of context-specific interventions to improve the nutritional status of women in rural Guatemala. The results describe key determinants and indicators of nutritional status, including anthropometry, dietary intake and diet quality. It should also be acknowledged that participants were recruited through a community health network, which may over-represent women actively engaged in support programs. This selection pattern could influence the dietary and anthropometric profiles observed, further reinforcing that the findings are descriptive of this specific group and not representative of the wider community.

Anthropometric measurements revealed a high prevalence of overweight and obesity (68.2% of participants), with more than 80% showing elevated waist-to-hip and waist-to-height ratios, indicating marked central adiposity. Altered fasting glycemia and hypertriglyceridemia in a subset of participants, combined with increased waist circumference, suggest a potential presence of metabolic syndrome [15,22]. However, these biochemical findings derive from a very small subsample (*n* = 11) and must therefore be interpreted with extreme caution. They provide only exploratory insight into possible metabolic risk patterns rather than allowing inference about prevalence in the broader community. Although similar cardiometabolic concerns have been documented in national reports among Guatemalan women [23], the present findings should be interpreted strictly within the context of the studied group and not as indicative of population-level burden.

The 24-hR indicated a caloric surplus, approximately 35% above estimated energy needs [20], which helps explain the high proportion of overweight and obesity observed in this specific group. Although macronutrient contributions (% of energy) fell within or slightly below recommended ranges, absolute intakes of carbohydrates, fats (especially saturated), and proteins were markedly elevated. High consumption of sugar-sweetened beverages and ultra-processed foods contributed to an energy-dense, nutrient-poor dietary pattern. While similar dietary concerns have been widely discussed in the Guatemalan nutrition literature [24,25,26], in the present study these patterns should be interpreted solely as characteristics of this convenience sample, without extrapolation to national dietary trends. From a micronutrient perspective, a high sodium intake and low intakes of calcium, potassium, and zinc [27,28] emerged. Although micronutrient deficiencies remain a public health concern in Guatemala [29], the present findings are descriptive and limited to this specific sample. The low dietary diversity observed, reflected by the MDD-W index, further suggests micronutrient vulnerability, although conclusions remain descriptive and limited to this convenience sample. Taken together, the coexistence of excessive energy intake and insufficient micronutrient intake observed here highlights a pattern of nutritional imbalance at the sample level, without implying broader population-level conclusions.

Women’s central role in rural food systems amplifies the relevance of understanding their nutritional status. As primary caregivers and household food managers, they influence meal composition and children’s diets, with implications for intergenerational health [30,31,32,33]. While these contextual factors are well-documented in the literature, the present study was not designed to evaluate structural determinants of the food environment. Therefore, broader interpretations related to food-system drivers are now presented cautiously and solely as background context, not as conclusions drawn from our data. In line with the small-scale observational nature of this study, possible implications for future interventions should be interpreted as suggestions rather than evidence-based recommendations. Community-based nutrition education could be explored as a potential avenue for addressing the excessive caloric intake and low nutrient density observed in this sample, particularly by focusing on reducing sugar-sweetened beverages and snack foods and promoting nutrient-dense options such as legumes, fruits and vegetables. Participatory approaches, such as cooking demonstrations and portion-size guidance, have shown promise in comparable settings [34,35]. Beyond individual behavior change, future research could investigate how local food environments influence dietary choices in this community. Strategies such as strengthening local markets, supporting home gardens or promoting traditional plant-based foods, frequently cited in the literature as culturally appropriate and cost-effective approaches [36,37], may warrant exploration in subsequent, larger studies. Any such considerations exceed the analytical scope of the present work and are proposed solely as potential directions for future investigation.

## 5. Limitations

This study has several limitations. First, its cross-sectional design precludes causal inference. Second, the small sample size (*n* = 22) and the non-probabilistic recruitment strategy limit the generalizability of the findings beyond the studied communities. Third, dietary intake was assessed using a single 24 h recall per participant, alongside a food frequency questionnaire (FFQ). While multiple non-consecutive 24 h recalls are recommended to capture intra-individual variability, logistical constraints and the unfunded, exploratory nature of this study made repeated assessments unfeasible. Both dietary assessment tools rely on self-report and are subject to measurement error, including misreporting and imprecision in portion size estimation.

Furthermore, individual energy and nutrient intakes were derived from household-level 24 h recalls using proportional allocation. This approach does not capture intra-household variation in food distribution and may introduce uncertainty, particularly in the estimation of micronutrient intakes. As a result, the derived values should be interpreted as approximate indicators of individual intake and dietary patterns rather than precise measurements. Additionally, although nutrient intake was estimated using the INCAP food composition tables, the potential influence of local food preparation methods, recipe variability, and ingredient heterogeneity could not be fully accounted for and may have affected nutrient estimations. Fourth, to allow comparison with mean energy intake derived from the 24 h recalls, energy requirements were estimated using a single reference value representing the average requirement of the study sample, rather than individual-specific needs. Consequently, the estimated energy surplus should be interpreted as an approximate indication at the sample level, and not as a precise measure of individual energy balance.

In addition, biochemical analyses were conducted in a subsample of participants (*n* = 11), limiting statistical power for these outcomes. Moreover, recruitment through the Cabrini Foundation community network may have resulted in a sample not fully representative of the broader population. Accordingly, this small-scale observational study was designed to generate preliminary insights rather than population-level estimates. Despite these limitations, the study provides novel preliminary data on dietary patterns, anthropometric status, and metabolic risk among women living in a rural–urban transitional area of Guatemala, highlighting the need for culturally sensitive, multisectoral interventions to improve diet quality and prevent metabolic risk.

## 6. Conclusions

This small-scale observational study provides a preliminary snapshot of dietary patterns and anthropometric characteristics among a sample of women from two rural communities in Guatemala. While the findings cannot be generalized beyond the study sample, they highlight nutritional features that may warrant further investigation, including limited dietary diversity and the coexistence of potential nutritional and metabolic vulnerabilities. These results are intended to inform the design of future, larger-scale studies and community-based nutrition education initiatives tailored to similar settings. Further research involving larger, probabilistic samples and more robust dietary assessment methods is needed to better characterize the double burden of malnutrition among adult women in rural Guatemalan contexts and to evaluate the impact of context-specific interventions.

## Figures and Tables

**Table 1:** Demographic characteristics of the study population.

	Study Population *n* = 22
**Age, years**	41.3 (16.3)
**Age range**	20–74 years
**Ethnicity**	
Ladina	21 (99%)
Maya	1 (1%)
**Educational level**	
Unschooled	1 (5%)
Elementary school	6 (27%)
Middle school	9 (41%)
High-school	6 (27%)
**Occupation**	
Housewife	21 (95%)
Secretary	1 (5%)
**Marital status**	
Married	10 (45%)
Cohabitant	10 (45%)
Single	1 (5%)
Widow	1 (5%)
**Household composition**	
2–3 people	5 (23%)
4–5 people	13 (59%)
More than 5 people	4 (18%)
**Children living in the household**	
None	2 (9%)
1–2 children	17 (77%)
3 children	3 (14%)

Categorical variables presented as absolute frequencies (*n*) and percentage (%), and continuous variables as mean (standard deviation). Ladina: mixed or non-indigenous background, primarily Spanish-speaking and culturally Westernized.

**Table 2:** Anthropometric characteristics of the study population.

	Study Population *n* = 22
**Anthropometric measurements**	
Weight, kg	63.9 (12.6)
Height, cm	149.5 (5.6)
BMI, kg/m^2^	29.0 (6.3)
Fat mass, %	37.6 (14.0)
Muscular mass, kg	34.6 (4.1)
Waist circumference, cm	95.6 (8.9)
Hip circumference, cm	98.3 (17.3)
WHtR	0.6 (0.1)
WHR	1.0 (0.2)
**BMI classes**	
Normal weight	7 (31.8%)
Overweight	5 (22.7%)
Obesity	10 (45.5%)
**Central adiposity**	
WHtR ≥ 0.59	19 (86%)
**Cardiometabolic risk**	
WHR ≥ 0.85	21 (95%)

Categorical variables presented as absolute frequencies (*n*) and percentage (%), and continuous variables as mean (standard deviation). Body Mass Index (BMI); Waist-to-height ratio (WHtR); Waist-to-hip ratio (WHR).

**Table 3:** Comparison between the mean intakes obtained from the 24-hR and the INCAP daily nutritional recommendations.

	Data from 24-hR *n* = 22		References Values from INCAP Daily Nutritional Recommendations
	Mean (SD)	Δ from Standard	Standard
**Energy (kcal)**	2450.2 (669.1)	↑	1900 kcal
**Macronutrients**			
Proteins (%)	12 (3.5)	↓	12%
Proteins (g)	74.3 (21.2)	↔	54.7 g
Total lipids (%)	34.4 (17.0)	↑	30%
Total lipids (g)	95.0 (46.3)	↑	63.3 g
Saturated fats (%)	6.6 (3.1)	↔	<10% total energy
Saturated fats (g)	19.8 (8.6)	↔	<21.1 g
Polyunsaturated fats (%)	7.2 (5.5)	↔	6–11% total energy
Polyunsaturated fats (g)	18.1 (15.0)	↑	12–23 g
Cholesterol (mg)	413.6 (243.0)	↑	<300 mg
Carbohydrates (%)	53.6 (15.0)	↓	58%
Carbohydrates (g)	332.5 (92.1)	↑	275.5 g
Fiber (g)	19.4 (10.3)	↓	30 g
**Micronutrients (minerals)**			
Calcium (mg)	508.3 (170.3)	↓	1000 mg
Sodium (mg)	2570.6 (1238.5)	↑	1500 mg
Potassium (mg)	1711.8 (622.6)	↓	4700 mg
Magnesium (mg)	295.3 (148.1)	↑	275 mg
Phosphorus (mg)	1380.1 (418.5)	↑	700 mg
Zinc (mg)	6.8 (2.9)	↓	14.6 mg
Iron (mg)	19.5 (6.3)	↑	15.6 mg
**Micronutrients (vitamins)**			
Vitamin A (IU)	474.3 (293.0)	↓	650 ng
Thiamin (mg)	1.4 (0.6)	↑	1.1 mg
Riboflavin (mg)	1.4 (0.3)	↑	1.1 mg
Niacin (mg)	14.0 (5.4)	↔	14 mg
Vitamin B6 (mg)	1.1 (0.5)	↓	1.3 mg
Vitamin B12 (µg)	3.2 (2.9)	↑	2.4 µg
Folic acid (µg DFE)	229.1 (133.7)	↓	400 µg EFD
Vitamin C (mg)	63.6 (40.4)	↓	65 mg

Data are presented as mean (standard deviation); international unit (IU); Dietary Folate Equivalents (DFE). INCAP daily nutritional recommendations [20]. ↓ = below the reference; ↑ = above the reference; ↔ = between ranges.

**Table 4:** Number of women consuming the different food groups in the MDD-W.

Food Groups Consumed	Total Sample *n* = 22
Grains, white roots and tubers, and plantains	22 (100.0%)
Pulses (beans, peas and lentils)	17 (77.3%)
Nuts and seeds	0 (0.0%)
Milk and milk products	8 (36.4%)
Meat, poultry and fish	20 (90.9%)
Eggs	14 (63.6%)
Dark green leafy vegetables	5 (22.7%)
Other vitamin A-rich fruits and vegetables	8 (34.6%)
Other vegetables	7 (31.8%)
Other fruits	9 (40.9%)

Data are presented as absolute frequencies (*n*) and percentage (%). Minimum Dietary Diversity Index for Women (MDD-W) [18].

**Table 5:** Biochemical of a subgroup of the population.

	Study Population *n* = 11
Blood glucose, mg/dL	99.9 (92.8; 113.0)
Total Cholesterol, mg/dL	196.0 (159.5; 212.0)
Triglycerides, mg/dL	158.0 (101.55; 298.5)
Seric creatinine, mg/dL	0.6 (0.6; 0.7)
Seric uric acid, mg/dL	5.3 (4.4; 5.8)
BUN, mg/dL	12.1 (9.3; 13.7)

Data are presented as median (25th and 75th percentile). Blood Urea Nitrogen (BUN).

## Data Availability

The raw data supporting the conclusions of this article will be made available by the authors on request.

## Abbreviations

The following abbreviations are used in this manuscript:

24-hR	24 h dietary recall
BMI	Body mass index
BUN	Blood Urea Nitrogen
DFE	Dietary Folate Equivalents
FFQ	Food Frequency Questionnaire
HbA1c	Glycated hemoglobin
HDL	High-density lipoprotein cholesterol
HP	Hip circumference
INCAP	Instituto de Nutrición de Centro América y Panamá
IU	International unit
LAF	Physical activity level
LDL	Low-density lipoprotein cholesterol
MDD-W	Minimum Dietary Diversity Index for Women
VLDL	Very-low-density lipoprotein cholesterol
WC	Waist circumference
WHR	Waist-to-hip ratio
WHtR	Waist-to-height ratio
